# Molecular Structure and Antioxidant Properties of Alkali Metal Salts of Rosmarinic Acid. Experimental and DFT Studies

**DOI:** 10.3390/molecules24142645

**Published:** 2019-07-21

**Authors:** Renata Świsłocka, Ewa Regulska, Joanna Karpińska, Grzegorz Świderski, Włodzimierz Lewandowski

**Affiliations:** 1 Department of Chemistry, Biology and Biotechnology, Bialystok University of Technology, Wiejska Street 45E, 15-351 Bialystok, Poland; 2 Institute of Chemistry, University of Bialystok, Ciolkowskiego Street 1K, 15-245 Bialystok, Poland

**Keywords:** rosmarinates, IR, Raman, NMR, DPPH, FRAP, DFT calculations

## Abstract

The molecular structure of alkali metal rosmarinates was studied in comparison to rosmarinic acid using FT-IR, FT-Raman, ^1^H and ^13^C NMR spectroscopy, as well as density functional theory (DFT) calculations. The B3LYP/6-311+G(d,p) method was used to calculate optimized geometrical structures of studied compounds, atomic charges, dipole moments, energies, as well as the wavenumbers and intensities of the bands in vibrational and NMR spectra. Theoretical parameters were compared to experimental data. Antioxidant activity was determined using two spectrophotometric methods: (i) Assessing the ability to scavenge 1,1-diphenyl-2-picrylhydrazyl (DPPH) stable radical and (ii) assay of antioxidant power of ferric ions reducing (FRAP). The linear correlations were found between HOMO–LUMO (highest occupied molecular orbital–lowest unoccupied molecular orbital) energy gap and the reducing power expressed as FRAP (R = 0.77) as well as between IC_50_ values (the ability of quenching DPPH radicals) and *Δν*_as-s_(COO) in IR spectra (differences between asymmetric and symmetric stretching vibrations bands) (R = 0.99). Photochemical properties of studied compounds were also evaluated. The influence of alkali metal on the electronic system of the rosmarinic acid molecule was discussed.

## 1. Introduction

**Figure 1:**
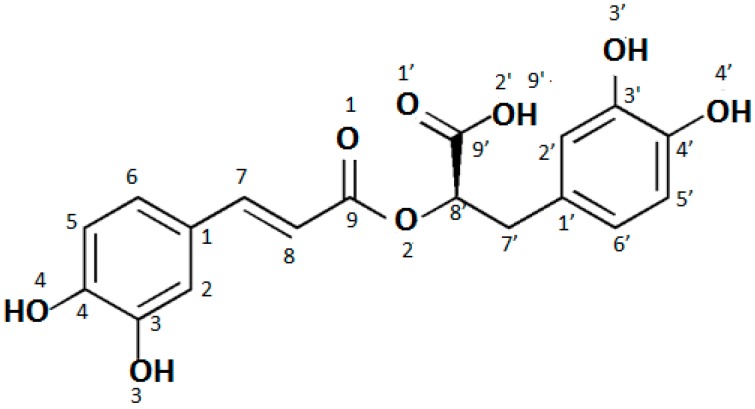
Numbering of atoms in rosmarinic acid molecule.

Rosmarinic acid is a naturally occurring bioactive compound. It is commonly found in plants such as rosemary, sage, lemon balm, oregano, lavender, mint, and savory [1,2,3,4]. The name of this compound comes from the plant from which rosmarinic acid was isolated for the first time (in 1958) [5]. Its structure was established as an ester of caffeic acid and 3,4-dihydroxyphenyllactic acid. The molecule of rosmarinic acid contains carboxylic group, two aromatic rings A and A’ with the ortho-catechol structures, the unsaturated C=C bond, and the ester moiety (Figure 1) [6,7]. Such a molecular structure entails specific properties of this compound. Rosmarinic acid has a lot of interesting biological activities. Its antioxidant activity is stronger than that of vitamin E [8] and it is the highest in the series of hydroxycinnamic acid derivatives: Rosmarinic acid > chlorogenic acid > caffeic acid > ferulic acid > coumaric acid [9,10]. The antimicrobial activity was also studied. Rosmarinic acid showed inhibitory and bactericidal exerts against some pathogenic bacteria, e.g., *Staphylococcus epidermidis*, *Staphylococcus lugdunensis*, *Stenotrophomonas maltophilia*, *Enterococcus faecalis*, *Pseudomonas aeruginosa*, *Corynebacterium*, *Mycobacterium smegmatis*, and *Staphylococcus warneri* [1]. The antiviral, antiallergic, neuroprotective, antiinflammatory, anti-HIV, and antitumor effects of rosmarinic acid were described in the literature [1,11]. According to Venkatachalam et al. [12] rosmarinic acid may be used as a potent chemopreventive agent in rat colon cancer. Kim et al. [1] reported that it might inhibit the metastasis of colorectal carcinoma. Properties [1,3], biosynthetic pathway [1,5], and the determination of rosmarinic acid in plant extracts [2,13,14] have already been described in the literature. Data concerning the molecular structure of rosmarinic acid [7,8] and its antioxidant activity [9,10] are also available, but there is no information about alkali metal rosmarinates. The influence of metal ions on the structure parameters as well as some physical, chemical, and biological properties of biological active molecules were previously studied by our team [15,16,17,18].

The aim of this paper is a spectroscopic study on molecular structure and antioxidant activity of alkali metal rosmarinates in comparison to rosmarinic acid. Obtained results are compared with theoretical data obtained by density functional theory (DFT) calculations.

## 2. Results and Discussion

### 2.1. Calculations

The density functional theory (DFT) calculations were performed using the Gaussian 09 [19] program running on PC computer and Gauss View [20] molecular visualization program. To calculate optimized geometrical structure of studied compounds the Lee-Yang-Parr Becke’s three parameter hybrid functional method (B3LYP) with 6-311+G(d,p) basis set was used. The optimized molecular geometry was characterized as minimum in the energy by the absence of imaginary wavenumbers.

The calculated lengths of the bonds and size of the angles of the optimized molecules of rosmarinic acid (RA) as well as lithium (RA-Li), sodium (RA-Na), and potassium (RA-K) rosmarinates are shown in Table 1. Results obtained for rosmarinic acid molecule were compared with calculated and experimental data found in the literature [7,21]. Good linear correlation was obtained for theoretical as well as experimental data. Correlation coefficients received for bonds’ lengths equal R = 0.9996 and 0.9997, respectively. Regarding the angles between bonds appropriate values are 0.9941 for the calculated and 0.9786 for the experimental one. Exemplary correlations are shown in Figure S1.

Analysis of the obtained data shows that salt is formed by the substitution of the alkali metal ion in place of the hydrogen cation in the carboxyl group of the rosmarinic acid molecule, since the biggest alternations were found within this group. The bond length between the O2′-H/M atoms in the salt molecules increases in comparison to the rosmarinic acid molecule from 0.894 Å to 1.562 Å (the numbering of atoms in the acid molecule is shown in Figure 1). Much smaller changes were noted in the bonds of C9′-O1′ (the increase by 0.056–0.062 Å) and C9′-O2′ (the decrease by 0.084–0.088 Å). In this way, these bonds in the COO group in salt molecules are almost equal. The lengths of bonds C9–O2, O2–C8′, and C8′-C9 change only by 0.004–0.017 Å. The remaining bonds in the studied molecules do not change at all. The size of the angles in the salt molecules also change a little compared to the acid molecule with the exception of the carboxyl group. The biggest changes were observed for C9′-O1′-H/M (the increase by 27.76–35.28°) and C9′-O2′-H/M (the decrease by 17.93–25.15°) angles. Much smaller changes were observed for the O1′-C9′-C8′ (the decrease by 6.94°), O1′-C9′-O2′ (2.09°) and C1′-C7′-C8′ (the increase by 0.97°) angles. The changes of angles around carbonyl group are about 1.2–1.3°. Other angles change only slightly (0.01–0.27°). This indicates, that the cation of metal is substituted instead of the hydrogen atom in the carboxyl group.

The lengths of bonds within the hydroxyl groups practically do not change in the series: RA → RA-Li → RA-Na → RA-K. On the other hand, the presence of weak intramolecular hydrogen bonds between the ortho-position hydroxyl groups should be noted. The bonds in acid molecule are similar to those in its salts molecules, so it is difficult to say, on this basis, which molecules will have stronger antioxidant properties, acid whether its salts. Antioxidant action of phenolic compound is based on free radical quenching. The free radical scavenging mechanism depends on hydrogen atom transfer (HAT), a single electron transfer (SET), or the combination of both HAT and SET, which may be described as ArOH → ArO^•^ + e^−^ + H^+^ [21]. Thus, antioxidant activity depends on (i) H-abstraction and (ii) stability of the formed free radical. Cao et al. [21] studied the geometry structures of ground state of rosmarinic acid and its free radicals formed on O3, O4, O3′, and O4′ atoms. They found that stronger intramolecular hydrogen bonds (1.97–1.98 Å) are in more radical form than in the ground state of acid molecule, which stabilizes the free radical forms and makes the abstraction of hydrogen atom from the hydroxyl group occur easily. The ability to create resonance semiquinone even quinone structures additionally results in the stability of the radicals.

By comparing two aromatic rings in studied molecules, it was found that the ring A’ has more equalized bonds lengths (ΔC′C′ = 0.013 $\dot{A}$) than the A ring (ΔCC = 0.023 $\dot{A}$) in acid as well as in salt molecules. This conclusion is confirmed when one compares the differentiation of angles. In the case of angles in the aromatic rings the variation in the size of the angles in the salt molecules (2.38° < ΔCCC < 2.55° and 2.55° < ΔC′C′C′ < 2.62°) is greater than in rosmarinic acid molecule (ΔCCC = 2.21° and ΔC′C′C′ = 2.40°). This is also visible in the values of aromaticity indices (Table 2) [22,23,24]. The ring A has probably more disturbed aromaticity because of the resonance semiquinone structures. On the other hand, A’ ring aromaticity is higher in salts molecules than in rosmarinic acid molecule, perhaps for the reason that the symmetry of the molecules increase.

The values of dipole moment, energy, and aromatic indices calculated for rosmarinic acid and its lithium, sodium, and potassium salts are shown in Table 2. The dipole moment values of rosmarinic acid and its salt molecules increase in the series: Rosmarinic acid < Li < Na < K salt. It indicates that polarity of studied compounds increase. The absolute energy of the tested molecules decreases in the same series. The values of the energy variation (ΔE) associated to the substitution reaction of the carboxylic hydrogen atom by an alkali metal atom have been calculated and included in Table 2.

**Figure 2:**
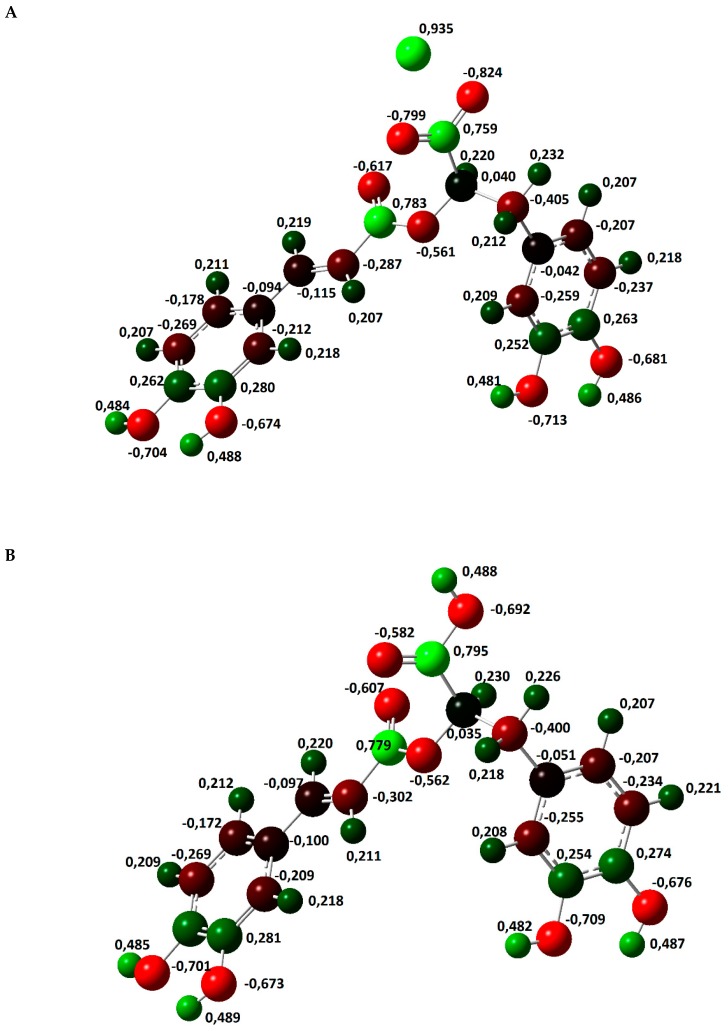
Optimized molecular structures of rosmarinic acid (**B**) and its sodium salt (**A**) as well as atomic charges.

**Figure 3:**
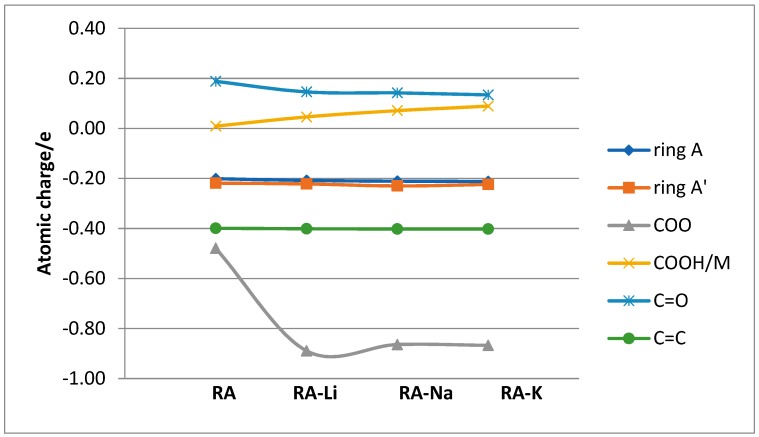
The atomic charges calculated for chosen groups of studied molecules.

The atomic charge distribution in studied molecules was calculated by the natural bond orbital method (NBO) using B3LYP/6-311+G(d,p). Data obtained for rosmarinic acid and its sodium salt are presented in Figure 2. In the case of lithium and potassium rosmarinates, the values of atomic charges are very similar to sodium salt. The atomic charges on most of the atoms in molecules of a lithium and potassium salts only slightly differ relative to the sodium rosmarinate molecule, they are in the range 0.000–0.007e. The exception are oxygen atoms of carboxylate group: O1′ (−0.813, −0.799 and −0.803e), O2′(−0.836, −0.824 and −0.822), as well as alkali metal ion (0.935, 0.935, and 0.956 e, for RA-Li, RA-Na, and RA-K, respectively). Systematic changes in the series: RA > RA-Li > RA-Na > RA-K is noted for atomic charges of C7, C8, and O1 atoms. When it comes to comparing salt molecules with an acid molecule, the largest changes are observed for H9′/M atom (atomic charge increases by 0.468e. In the case of O1′ and O2′ atoms, negative atomic charges significantly increase for lithium salts (by 0.231 and 0.144e, respectively) in comparison to acid, but in the Li > Na > K salts series they slightly decrease (0.014 and 0.012e). Much smaller changes (0.037e) in the series RA > RA-Li > RA-Na > RA-K occur for the charges on C9′ atom of carboxylate group. The summary charges calculated for the aromatic rings, double bond, and carbonyl and carboxylic/carboxylate groups were calculated and are shown in Figure 3. In summary, the biggest changes in comparison to acid molecule are observed for COO group. However, very good linear correlation was found between the summary charge calculated for the COOH/M group and electron affinity, the ionic potential, and the atomic radius of the hydrogen/metal atom. Corresponding correlation coefficients are 0.9998, 0.9918, and 0.9891, respectively.

**Figure 4:**
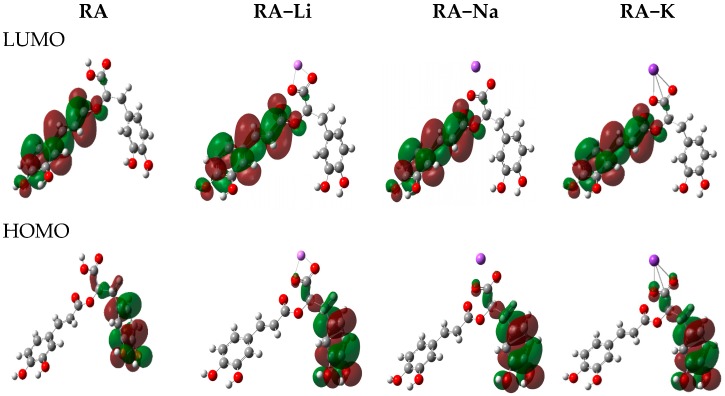
The HOMO/LUMO (highest occupied molecular orbital/lowest unoccupied molecular orbital) electron densities in molecules of studied compounds.

The frontier orbital energies are the important parameters of the molecular electron structure. The highest occupied molecular orbital (HOMO) and the lowest unoccupied molecular orbital (LUMO) energies as well as other general reactivity descriptors [25,26,27,28] such as ionization potential (I), electron affinity (A), electronegativity (*χ*), chemical hardness (*η*), softness (s), chemical potential (*μ*), electrophilicity (*ω*), and nucleophilicity (N) indexes calculated on the basis of the HOMO and LUMO orbital’s energy are gathered in Table 3. Reactivity descriptors were calculated as follows: I = −E_HOMO_; A = −E_LUMO_; *χ* = (I + A/2); *η* = (I − A)/2; s = 1/2*η*; *μ* = −(I + A)/2; *ω* = *μ*^2^/2*η*; N = E_HOMO(nuclefil)_−E_HOMO(**TCNE**)_ (nucleofilicity index N is referred to tetracyanoethylene TCE [28). The highest occupied molecular orbital (HOMO) and the lowest unoccupied molecular orbital (LUMO) play an important role for predicting the charge transfer within the molecule, chemical reactivity, bioactivity, and stability of the compound [25,29]. The higher the HOMO energy is, the stronger electron donor molecule is, while LUMO energy reflects the ability to accept the electron. The distribution of HOMO electron density in a phenolic molecule may qualitatively indicate the active site of scavenging free radicals, because the reaction of H-abstraction is associated with the transfer of electron [21]. The HOMO and LUMO electron densities for rosmarinic acid and its lithium, sodium, and potassium salts are shown in Figure 4. The HOMO electron density in both acid and its salt molecules is mainly distributed over carbon atoms in A’ ring and O3′, O4′ oxygen atoms of ortho-hydroxyl groups, which can donate electrons easily during the H-abstraction. The LUMO electron density is distributed over ring A, double bond, carbonyl and carboxylic groups forming a conjugating system. This is beneficial for electron scattering and has some effects on free radicals such as O_2_^−^ [21]. The HOMO-LUMO energy gap is a useful descriptor of chemical and biological activity of molecules. The smaller gap value is, the more chemically active the molecule is, and it is termed a soft molecule. The HOMO-LUMO gap values decrease in the following order: RA-Li > RA-Na > RA-K > RA, indicating the increase in the reactivity of studied molecules in above series. In this way rosmarinic acid should be chemically more active then salts molecules. The electrophilic or nucleophilic index is related to molecule ability to exchange electron density during a reaction [30,31]. The electron affinity, electronegativity and electrophilicity indexes decrease in series: rosmarinic acid > its lithium > sodium > potassium salts, while the values of chemical potential increase in the above series. The ionization potential decreases in order RA-Li > RA > RA-Na > RA-K, whereas the values of nucleophilicity increase in the above series. It indicates that the sodium and potassium salts have higher electron transfer ability than rosmarinic acid.

The wavenumbers and intensities of bands in vibrational spectra for the optimized structures of rosmarinic acid and its lithium, sodium, and potassium salt molecules were also calculated and presented in Table 4.

### 2.2. Vibrational Spectra

**Figure 5:**
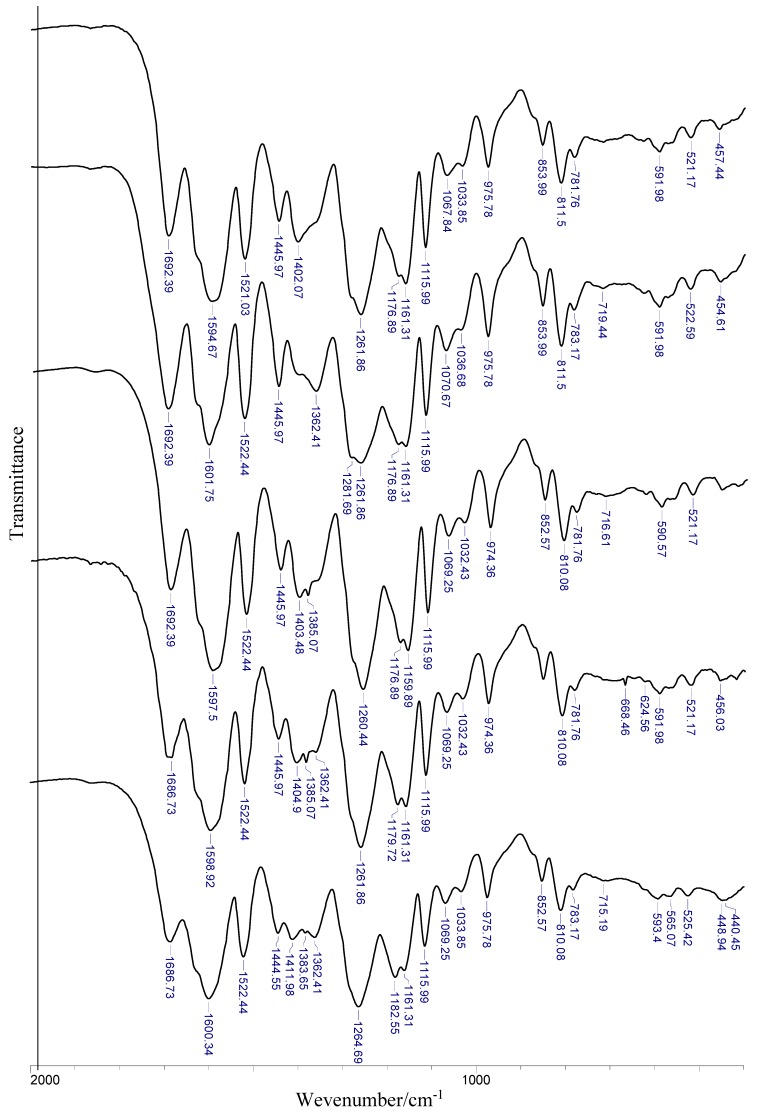
Experimental vibrational spectra of Li, Na, K, Rb, and Cs rosmarinates (shown from bottom to top).

Experimental data of IR and Raman spectra of rosmarinic acid and its alkali metal salts are gathered in Table 5 and visualized in Figure 5. The assignment of bands is based on the literature, quantum-mechanical calculations, and personal experience. The bands are numbered along with the notation used by Varsányi [32].

In the IR spectrum of rosmarinic acid, there is a very intense band originating from the stretching vibration of the carboxyl group νC=O at 1708 cm^−1^, while in Raman there are two bands of νC=O at 1708 and 1727 cm^−1^ (weak and medium intensity, respectively). In the rosmarinic acid spectrum the bands associated with in plane βC=O at 790 cm^−1^ (IR) and 792 cm^−1^ (Raman) as well as out of plane γC=O at 682 cm^−1^ (IR) deformations are observed. Some bands are connected with vibrations of hydroxyl groups, they are weak and of medium intensity bands coming from the stretching vibrations νOH at 3419, 3456 and 3399 cm^−1^ (IR) and deformations βOH at 1478 cm^−1^ and γOH at 917 cm^−1^ (Raman). In addition, a number of bands associated with vibrations of the aromatic ring can be seen in the ligand spectrum.

The substitution of the alkali metal atom in place of the hydrogen atom in the carboxylic group causes the disappearance of some characteristic for acid bands in the salt spectra, i.e., the bands coming from stretching and deformation vibrations of carboxylic group. Instead, there are very intense bands derived from the asymmetric stretching vibrations of carboxylate anion ν_as_(COO^−^) lying in the range 1608–1606 cm^−1^ (Raman) and 1600–1598 cm^−1^ (IR) and symmetrical stretching vibration bands ν_s_(COO^−^) in the range 141–1403 cm^−1^ (IR spectra). The less intense bands derived from symmetrical in plane deformations β_s_(COO^−^) appear at 738–736 cm^−1^ in IR spectra (751–728 cm^−1^. In Raman) and asymmetrical deformations β_as_(COO^−^) in the range 565–564 cm^−1^ (IR) and 573–568 cm^−1^ (Raman) as well as out of plane deformations γ_s_(COO^−^) bands (715–718 and 724–713 cm^−1^, respectively in IR and Raman spectra). There are also low-intensity bands in Raman spectra, for example bands of symmetrical stretching vibrations ν_s_(COO^−^) in the range 1417–1407 cm^−1^. The Raman spectra of alkali metal rosmarinates show a decrease in the wavenumbers and a reduction in the intensity of the bands coming from the vibrations of the aromatic ring, e.g., ν_as_(CH_2_), β(CH), 19b (βOH, νCC), 16b (ϕCC), and 6b (αCCC) in comparison to the acid spectrum. In addition, the wavenumbers of some bands change regularly in the series of Li → Cs salts. The bands of β_s_(COO^−^) in Raman spectra, ν_s_(COO^−^) and ν_as_(COO^−^) in IR spectra are shifted to lower wavenumbers in Li > Na > K > Rb > Cs rosmarinates series but the bands of β(CH) deformation in the range 1075–1068 cm^−1^ and 5 (γCH) in Raman spectra show an increasing tendency in the above series. However a systematic decrease of the wavenumbers of the bands of asymmetric and symmetric stretching vibrations of carboxylate anions in the IR spectra was observed in the Li > Na > K > Rb > Cs salt series, the values of difference Δν = ν_as_(COO^−^) − ν_s_(COO^−^) increase in the order: Li > Cs > Na > K > Rb, correspondingly 188, 193, 194, 195, and 203 cm^−1^. It indicates an increasing share of metal-oxygen ionic binding.

The very good linear correlation was observed between the values of wavenumbers of chosen bands (ν_s_(COO), β_s_(COO), β(CH), 5) and the parameters characterizing metals, i.e., electronegativity, atomic radius, electron affinity, and ionic potential (defined as the ratio of the ion charge to its radius). For electronegativity, ionic potential, and atomic radius, high correlation coefficients (0.875–0.987) were obtained. In addition, it was found that the wavenumbers of ν_s_(COO^−^) band very well linearly correlates with the lengths of bonds (C9′-O1′ and C9′-O2′) and sizes of angles (O1′-C9′-O2′, C9′-O1′-M and C9′-O1′-M) of carboxyl group (correlation coefficients are in the range 0.991–0.999).

**Figure 6:**
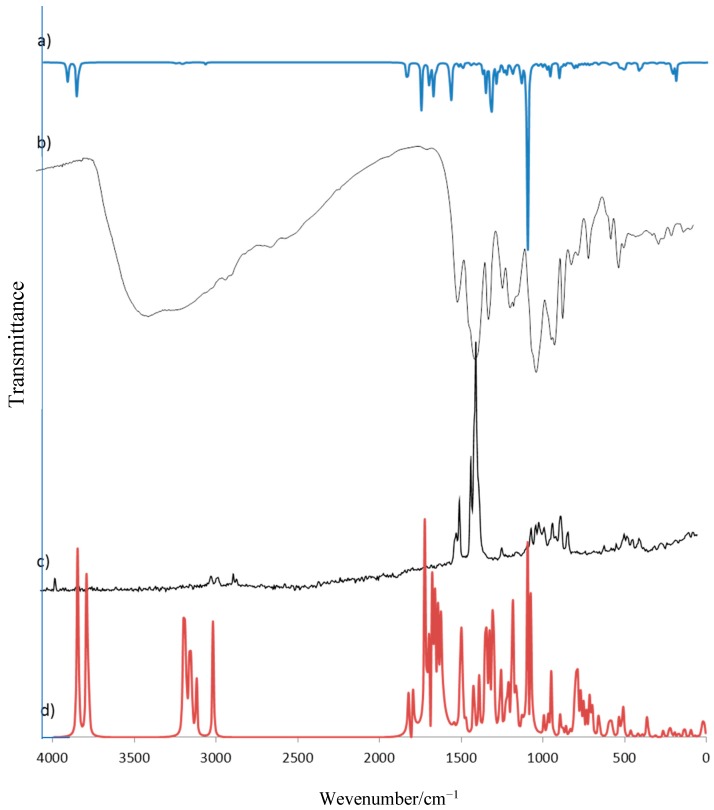
Experimental and calculated vibrational spectra of rosmarinic acid: FT-IR (**a**,**b**) and Raman (**c**,**d**).

Experimental data have been compared to calculated wavenumbers and the good linear correlation is found (correlation coefficient R = 0.998, 0.999, 0.998, and 0.999, respectively for RA, RA-Li, RA-Na, and RA-K). Experimental and calculated vibrational spectra are presented exemplary for rosmarinic acid in Figure 6.

### 2.3. NMR Spectra

The experimental and theoretical chemical shifts of protons and carbons in NMR spectra of rosmarinic acid and its alkali metal salts in DMSO saturated solution are gathered in Table 6. Literature data [8] of experimentally obtained chemical shifts in ^13^C and ^1^H NMR spectra of rosmarinic acid are also presented. The good correlation between literature and the obtained data in this work is found (R = 0.999 for ^13^C as well as ^1^H NMR).

The good linear correlation is also obtained between our experimental and calculated data connecting NMR spectra obtained for rosmarinic acid and its lithium, sodium, and potassium salts is obtained. The correlation coefficients equal 0.857, 0.879, 0.880 and 0.874 for ^13^C NMR, for ^1^H NMR corresponding values are: 0.987, 0.976, 0.979, and 0.938.

In ^13^C NMR spectra there are peaks connecting carbons of aromatic rings (C1–C6) and (C1′-C6′), the double bond (C7–C8), the ester group C9 and carboxyl group C9′. ^13^C NMR spectra of rosmarinic acid and its alkali metal salts shows the highest values of chemical shifts for C9′ carbon atom (170.8–173.1 ppm), slightly lower values of chemical shifts are observed for C9 atom (165.9–166.3 ppm). The chemical shifts observed for C3, C4, and C3′, C4′ atoms (the places of hydroxy groups substitution) as well as for C7 atom (of double bond) are in the range 143.7–148.9 ppm. The lowest chemical shift is observed for C7′ atom of CH_2_ group (36.1–37.3ppm). The chemical shifts for C8′ carbon atom are 72.8–76.2 ppm. The highest changes in salt spectra in comparison to acid spectrum is noted for C8′ and C1′ (127.3–129.9 ppm) carbon atoms. The increase in chemical shifts in salts spectra in comparison to acid spectrum is observed in the case of C2, C9, C1′, C2′, C7′, C8′, and C9′ atoms. In comparison, a decrease is noted for C6, C7, C3′, and C4′ atoms. There are no regular changes in the series of alkali metal rosmarinates in the experimental ^13^C NMR spectra. In theoretical spectra the decrease in chemical shifts is noted for C2, C3, C4, C5, C6, C9, C3′, C5′, C6′, and C9′, but only an increase for C1 and C1′ atoms.

In calculated ^1^H NMR spectra of rosmarinic acid and its alkali metal salts the chemical shifts for H3, H4, and H3′, H4′ protons are in the range 4.3–5.5 ppm. The signals for above atoms in experimental spectrum of acid are in the range 8.7–9.6 ppm, but in salts spectra corresponding signals are not found. The phenolic proton signals is usually a sharp singlet in the range of 7.5–4.0 ppm. The presence of two groups relative to each other in the ortho position strongly influences the chemical shift of phenolic protons (signals are in the range of 12–10 ppm). This is due to the formation of intramolecular hydrogen bonds [33]. In calculated spectra, the peak assigned for H9′ proton of carboxylic group occurs at 6.0 ppm, whereas in experimental spectra it is found at 13.0 ppm. Similar results were obtained in our previous investigations [27]. In experimental ^1^H NMR spectra of rosmarinates, the decrease in chemical shifts in comparison to acid spectrum is observed in the case of H6, H7, H8, H5′, H6′, H7′, and H8′ protons, while the increase is only noted for H7′. In theoretical spectra the decrease is observed for H3, H4, H3′, H4′, H8, H2′, H5′, and H6′ protons, and the increase for H7″ and H7 atoms. When it comes to comparing spectra of alkali metal rosmarinates, in experimental spectra, only slight changes are noted, while in theoretical spectra, the decrease in the series Li > Na > K salts is observed for the most protons, the increasing tendency is only observed for H7′.

The linear correlation was found between chemical shifts of C9′ atom in experimental NMR spectra and some metal parameters such as electronegativity (R = 0.773), ionic potential (0.766), atomic radius (0.762), and electron affinity (0.708).

### 2.4. Antioxidant Activity

**Figure 7:**
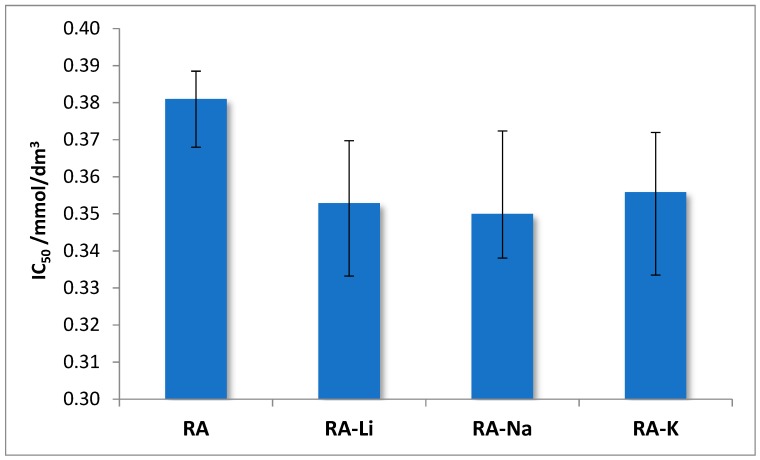
The IC_50_ values obtained in DPPH analysis for rosmarinic acid (RA) and its lithium, sodium, and potassium salts.

The antioxidant properties of rosmarinic acid and its salts with lithium, sodium, and potassium were examined. To assess the antioxidant activity, two methods were used that consist of (i) scavenging of free, stable DPPH* radicals and (ii) reduction of Fe^3+^ ions (FRAP method). The obtained results connecting the DPPH radical scavenging activity of studied compounds are expressed as IC_50_. The lower IC_50_ value, the better DPPH radical neutralizing properties represented by the test samples. Studies show that alkali metal rosmarinates have better antioxidant properties than rosmarinic acid, IC_50_ values obtained for salts are very similar (Figure 7).

The clear linear correlation was found between differences of wavenumbers *Δν*_as-s_(COO) in IR spectra and IC_50_ values (effective concentration of the solution required to decrease the initial DPPH radicals by 50%). The correlation coefficient equals 0.989.

**Figure 8:**
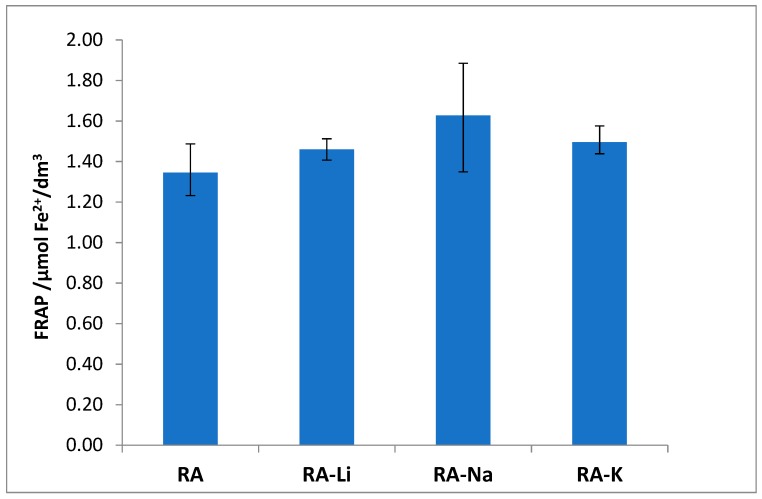
Reducing power of the rosmarinic acid (RA) and its lithium, sodium, and potassium salts evaluated by FRAP (Fe^3+^ ion reduction capability) assay.

The studied salts have also higher ability to Fe^3+^ reduction than rosmarinic acid (Figure 8), and among them the sodium rosmarinate shows the highest antioxidant activity.

The antioxidant properties expressed through the Fe^3+^ ion reduction capability (FRAP method) linear correlate with the values of HOMO-LUMO energy gap. The correlation coefficient equals 0.7666.

### 2.5. Photochemical Properties

The results of kinetics studies of direct photolysis of rosmarinic acid and its salts are collected in Table 7. The first order kinetics model was assumed. Based on the obtained results, it could be stated that rosmarinic acid salts, as well as it alone, are photoresistant substances. The half times of the process are in the range 14.4 h (for rosmarinic acid) to 5.3 h (for sodium rosmarinate). This observation is in good agreement with literature data [34]. The 5 h exposition of the trans-RA solution in THF to the daylight resulted in lowering of its content by 2.5%. After a period of one month of continuous exposure to light, a loss of 22.3% of the amount of RA was found [34]. It was stated that under influence of light aphoto-isomerization process occurred and cis-RA is the only product of degradation [34]. A slight increase in pH values for rosmarinic acid and its salts solutions were observed after 2 h of irradiation with the exception of cesium and lithium rosmarinates (decrease).

The linear correlation between reaction rates and energy gap between HOMO and LUMO levels are observed in the series RA → RA-Li → RA-Na (R = 0.730).

## 3. Experimental Section

### Materials and Methods

The alkali metal salts of rosmarinic acid were prepared by mixing in a 1:1 stoichiometric ratio mixture of rosmarinic acid and the standard solution of appropriate hydroxide with a concentration of 0.1 mol/L. The obtained mixture was dissolved in an ultrasonic bath. The water was evaporated in a water bath. The obtained precipitates were dried at 105 °C for 24 h. Rosmarinic acid, hydrochloric acid, sodium carbonate, as well as lithium, sodium, potassium, rubidium, and cesium hydroxides were from Sigma-Aldrich.

FT-IR and FT-Raman spectra of rosmarinic acid and synthesized salts were performed by method of pressing the sample within KBr. The spectra were recorded in the range of 400–4000 cm^−1^ at a resolution of 4 cm^−1^.

The NMR spectra of DMSO saturated solution were recorded with the NMR AC 200 F, Bruker unit at room temperature. TMS was used as an internal reference.

The determination of antioxidant activity was done by measuring the ability to quench the synthetic DPPH (1,1-diphenyl-2-picrylhydrazyl) radical by a modified Brand-Williams method [35]. For this purpose, the aqueous solutions of the tested compounds at the appropriate concentrations were prepared. Then, 0.05 mL of each sample was added to 2.5 mL of a methanolic solution of DPPH (absorbance is approx. 1.0). The samples were incubated in a dark at room temperature for 30 min. Absorbance of solutions was measured at λ = 515 nm against the blind test. All measurements were made in six replications. The percentage of the reduction of DPPH radical after incubation with the test solutions with reference to the control sample was calculated from the below equation:

$$DPPH\;\left[\%\right]=\;\frac{A_{0}-A_{s}}{A_{0}}\times 100\%$$

where *A*_0_—absorbance of control sample and *A_S_*—absorbance of tested sample. The results were expressed as inhibitory concentration at 50% (IC_50_), which is the concentration of the test solution for achieving 50% of the radical scavenging capacity.

The determination of the ability to reduce iron in Fe^3+^-TPTZ complex (ferric-2,4,6-tripyridyl−s-triazine) into the Fe^2+^−TPTZ complex by the test compounds was made according to the FRAP method described by Benzie and Strain [36]. 0.05 mL of the samples was added to 2.5 mL FRAP reagent and incubated at room temperature for 15 min and then the absorbance of the solution was measured at λ = 596 nm and converted to the μmol Fe^2+^/L based on a comparison of absorbance values of samples with absorbance of Fe^2+^ standard measured under the same conditions. The FRAP reagent was prepared by mixing acetate buffer (pH 3.6), TPTZ (0.01 M), and FeCl_3_ (0.02 M) in a 10:1:1 ration, respectively.

To determine the antioxidant capacity of studied compounds following reagents were used: DPPH and FeCl_3_ from Sigma Aldrich; TPZZ from Fluka; methanol from Merck; HCl, CH_3_COONa from Chempur.

Evaluation of photochemical properties of rosmarincic acid and its salts was done as follows. A 50 mL working solution of currently studied solution at the concentration 20 µg/mL was subjected to the irradiation in a solar light simulator chamber SUNTEST CPS+, ATLAS USA equipped with a xenon lamp emitting radiation in the 300–800 nm range with an intensity of 500 Wm^−2^ for two hours. Illumination was stopped every 15 min, 2 mL of solution were taken and absorption spectrum was recorded. The absorbance changes were read at 324 nm as an analytical wavelength. All applied for photochemical experiments solutions were prepared by dissolving of the appropriate weighted mass of the studied substance in 100 mL of MiliQ water. All samples tested were prepared in triplicate.

Quantum mechanical calculations for rosmarinic acid and its salts molecules were made using the B3LYP method with the 6-311+(d,p) basis set and the GAUSSIAN 09 package of programs [19] running on a PC computer. Visualization of calculated parameters was performed by GaussView molecular visualization program [20]. The optimized molecular geometry was characterized as minimum in the energy by the absence of imaginary wavenumbers. To make quantity evaluation of aromaticity various indexes were calculated: A_J_: normalized function of variance of bond lengths in the perimeter of the molecule; BAC: bond alternation coefficient; HOMA: abbreviation from Harmonic Oscillator Model of Aromaticity (HOMA = 1 − EN − GEO); GEO: geometric contribution to the aromaticity; EN: energetic contribution to the aromaticity; I_6_: Bird’s indice, which describes the geometric contribution to the aromaticity. The values of energy, dipole moment, atomic charges, general reactivity descriptors, as well as the wavenumbers and intensities of bands in vibrational spectra were calculated.

## 4. Conclusions

Spectroscopic study on alkali metal rosmarinates in comparison to rosmarinic acid molecule has been presented. The molecular structure has been determined using DFT calculation as well as the experimental spectroscopic techniques. Analysis of the obtained data shows that salt is formed by the substitution of alkali metal atom instead of the hydrogen atom in the carboxyl group, since the biggest changes in alkali metal rosmarinates in comparison to acid molecule regarding bond lengths, size of angles, and atomic charges were found. The substitution of the alkali metal atom in place of the hydrogen atom in the carboxylic group was confirmed by experimental as well as calculated vibrational spectra. The disappearance of some characteristic for acid bands in the salt spectra, i.e., the bands coming from stretching and deformation vibrations of carboxylic group was observed. In the IR or Raman spectra, the systematic decrease or increase of the wavenumbers of the bands not only of carboxylate anion was observed in Li → Na → K → Rb → Cs rosmarinates series. The increase in Δν = ν_as_(COO^−^) − ν_s_(COO^−^) difference in the above series indicates an increasing share of metal-oxygen ionic binding.

The very good linear correlation was observed between the values of wavenumbers of some bands in IR and Raman spectra and the parameters characterizing metals. The linear correlations were obtained for electronegativity, ionic potential, and atomic radius (correlation coefficients are in the range 0.88–0.99). In addition, it was found that the wavenumbers of ν_s_(COO^−^) vibrations band very well-linearly correlate with the lengths of bonds and size of angles of the carboxyl group (0.99 < R < 1.00).

A very good linear correlation was found between the summary charge calculated for the COOH/M group and electron affinity, the ionic potential and the atomic radius of the hydrogen/metal atom (0.99 < R < 1.00).

The dipole moment values of rosmarinic acid and its salts molecules increase in the series: Rosmarinic acid < Li < Na < K salt, it follows that the polarity of studied compounds increase. The HOMO-LUMO energy gap is useful descriptors of chemical and biological activity of molecule. The smaller is gap value, the more chemically active the molecule is. Presented data in this manuscript show that rosmarinic acid should be chemically more active than salt molecules, while in the series of salts, the reactivity increases. Whereas the study on antioxidant activity of rosmarinic acid and its alkali metal salts indicate that alkali metal rosmarinates are better antioxidants than rosmarinic acid, the highest antioxidant properties have been found for sodium salt.

The antioxidant properties expressed through the Fe^3+^ ion reduction capability (FRAP method) linear correlate with the values of HOMO-LUMO energy gap (R = 0.77). On the other hand, IC_50_ values indicate the ability of quenching DPPH radicals correlate very well with *Δν*_as-s_(COO) in IR spectra (R = 0.99).

On the basis of the photochemical tests it was found that the rosmarinic acid and its alkali metal salts are photoresistant substances. The linear correlation between reaction rates and energy gap between HOMO and LUMO levels are observed in the series RA → RA-Li → RA-Na (R = 0.73).

## Figures and Tables

**Table 1:** The distances between atoms (Å) and the angles between bonds (°) in rosmarinic acid and its lithium, sodium, and potassium salt molecules calculated using B3LYP/6-311+G(d.p).

Atom Numbers ^1^	Rosmarinic Acid (RA)				RA-Li	RA-Na	RA-K
	Exp. [7]	Calc. [7]	Calc. [21]				
	The Distances between Atoms/Å ^2^						
C1-C2	1.395	1.411	1.393	1.407	1.407	1.407	1.407
C2-C3	1.377	1.385	1.392	1.383	1.384	1.384	1.384
C3-C4	1.384	1.407	1.412	1.406	1.406	1.406	1.405
C4-C5	1.386	1.396	1.383	1.388	1.388	1.387	1.387
C5-C6	1.372	1.390	1.413	1.393	1.392	1.393	1.393
C1-C6	1.391	1.407	1.406	1.403	1.403	1.402	1.402
C1-C7	1.462	1.457	1.457	1.459	1.461	1.462	1.462
C7-C8	1.317	1.348	1.350	1.344	1.343	1.342	1.342
C8-C9	1.459	1.469	1.469	1.470	1.475	1.477	1.478
C9-O1	1.210	1.219	1.218	1.210	1.211	1.212	1.213
C9-O2	1.327	1.363	1.365	1.367	1.358	1.354	1.350
O2-C8′		1.442	1.428	1.427	1.434	1.438	1.441
C1′-C2′		1.405	1.405	1.400	1.400	1.401	1.400
C2′-C3′		1.390	1.388	1.388	1.388	1.388	1.388
C3′-C4′		1.405	1.406	1.401	1.400	1.400	1.400
C4′-C5′		1.392	1.391	1.389	1.389	1.389	1.389
C5′-C6′		1.397	1.397	1.394	1.394	1.395	1.395
C1′-C6′			1.399	1.397	1.397	1.397	1.397
C1′-C7′		1.517	1.514	1.512	1.512	1.512	1.512
C7′-C8′		1.544	1.546	1.541	1.538	1.536	1.535
C8′-C9′		1.532	1.524	1.528	1.532	1.541	1.544
C9′-O1′		1.209	1.206	1.201	1.263	1.259	1.257
C9′-O2′		1.351	1.358	1.354	1.270	1.266	1.264
O2′-H/M		0.972		0.969	1.863	2.217	2.531
C3-O3	1.374	1.357	1.357	1.362	1.363	1.363	1.363
O3-H3	0.820	0.970	0.973	0.966	0.966	0.966	0.966
C4-O4	1.358	1.357	1.376	1.372	1.374	1.375	1.375
O4-H4	0.820	0.965	0.969	0.963	0.962	0.962	0.962
O4-H3		2.124	2.112	2.152	2.152	2.151	2.151
C3′-O3′		1.379	1.379	1.379	1.380	1.381	1.381
O3′-H3′		0.965	0.969	0.962	0.962	0.962	0.962
C4′-O4′		1.364	1.364	1.364	1.366	1.366	1.367
O4′-H4′		0.969	0.973	0.966	0.966	0.966	0.966
O3′-H4′		2.130	2.124	2.155	2.154	2.153	2.153
	**The Angles/°**						
C1-C2-C3	121.3	120.9		120.90	120.93	120.94	120.97
C2-C3-C4	119.8	120.3		119.69	119.72	119.73	119.73
C3-C4-C5	119.4	119.3		120.22	120.18	120.16	120.15
C4-C5-C6	120.6	120.3		119.77	119.78	119.79	119.79
C5-C6-C1	120.7	121.1		120.93	120.97	120.98	120.99
C2-C1-C6	118.1	118.1		118.49	118.42	118.40	118.37
C1-C7-C8	128.6	128.1		127.82	127.86	127.76	127.74
C7-C8-C9	121.5	119.8		120.28	120.44	120.60	120.71
C8-C9-O1		126.4		126.86	126.18	125.83	125.63
O1-C9-O2		122.8		122.63	123.38	123.84	123.97
C1′-C2′-C3′				120.58	120.65	120.66	120.70
C2′-C3′-C4′				120.64	120.67	120.71	120.71
C3′-C4′-C5′				119.09	119.05	119.03	119.02
C4′-C5′-C6′				120.16	120.16	120.15	120.16
C5′-C6′-C1′				121.11	121.18	121.26	121.26
C2′-C1′-C6′				118.43	118.29	118.20	118.16
C1′-C7′-C8′				113.35	113.82	114.03	114.32
C7′-C8′-C9′				110.67	110.73	111.22	110.86
C8′-C9′-O1′		125.2		126.17	120.90	119.69	119.23
O1′-C9′-O2′		123.1		123.58	122.17	124.79	125.67
C9′-O2′-H/M		105.9		107.41	82.26	87.08	89.48
C9′-O1′-H/M				54.55	82.31	87.23	89.83

^1^ the numbering of atoms according to Figure 1; ^2^ 1 Å = 10^−10^ m.

**Table 2:** Values of dipole moment, energy as well as aromaticity indices calculated using B3LYP/6-311+G(d.p) method for rosmarinic acid and its lithium, sodium, and potassium salts.

	Rosmarinic Acid	Rosmarinates		
		RA-Li	RA-Na	RA-K
Energy/Hartree ^1^	−1297.1	−1304.7	−1459.5	−1897.1
Energy variation ΔE ^2^/kJ/mol		−7351.4	−3675.7	−3675.7
Dipole moment/Debye (D) ^3^	4.480	6.736	8.457	10.207
**Aromaticity indices [22,23,24]**				
HOMA ^4^ Ring A	0.9576	0.9580	0.9587	0.9576
HOMA Ring A’	0.9828	0.9849	0.9840	0.9840
I_6_^5^ Ring A	92.0475	92.2489	92.1176	92.0475
I_6_ Ring A’	96.2393	96.6195	96.4861	96.4861
A_j_^6^ Ring A	0.9897	0.9902	0.9899	0.9897
A_j_ Ring A’	0.9977	0.9981	0.9980	0.9980
BAC ^7^ Ring A	0.8588	0.8628	0.8608	0.8588
BAC Ring A’	0.9313	0.9405	0.9385	0.9385

^1^ Hartree = 2625.5 kJ/mol; ^2^ ΔE = 2E(RA − M) + E(H_2_) − 2E(M) − 2E(RA); ^3^ 1 D = 3.3356∙10^−30^ C∙m; ^4^ HOMA—Harmonic Oscillator Model of Aromaticity; ^5^ I_6_—Bird’s Index; ^6^ A_j_—Julg’s Index; ^7^ BAC-Bond Alternation Coefficient.

**Table 3:** Energy of HOMO/LUMO orbitals and other reactivity descriptors.

Molecular Descriptor	RA	RA-Li	RA-Na	RA-K
HOMO energy/eV	−5.774	−5.788	−5.641	−5.558
LUMO energy/eV	−2.170	−1.871	−1.746	−1.698
HOMO-LUMO energy gap/eV	3.604	3.917	3.894	3.860
Electron affinity (A)/eV	2.170	1.871	1.746	1.698
Ionization potential (I)/eV	5.774	5.788	5.641	5.558
Chemical hardness (*η*)/eV	1.802	1.959	1.947	1.930
Chemical softness (s)/eV	0.277	0.255	0.257	0.259
Chemical potential (*μ*)/eV	−3.972	−3.830	−3.693	−3.628
Electronegativity (*χ*)/eV	3.972	3.830	3.693	3.628
Electrophilicity index (*ω*)/eV	4.378	3.744	3.503	3.410
Nucleophilicity index (N)/eV	3.713	3.699	3.847	3.930

I = −E_HOMO_; A = −E_LUMO_; χ = (I + A/2); η = (I − A)/2; s = 1/2η; μ = −(I + A)/2; ω = μ^2^/2η; N = E_HOMO(nuclefil)_ − E_HOMO(TCE)_; TCE−tetracyanoethylene.

**Table 4:** Calculated wavenumbers (cm^−1^) and intensities (Int) of bands in rosmarinic acid and its lithium, sodium and potassium salts spectra.

Rosmarinic Acid		Lithium Rosmarinate		Sodium Rosmarinate		Potassium Rosmarinate		Assignment *	
cm^−1^	Int	cm^−1^	Int	cm^−1^	Int	cm^−1^	Int		
3851	70.0	3852	64.1	3849	63.5	3852	65.0	νOH	
3846	144.8	3844	140.8	3848	149.5	3849	135.4	νOH	
3793	115.9	3796	34.3	3796	97.1	3795	104.4	νOH	
3792	116.2	3791	206.6	3794	130.2	3794	119.8	νOH	
3759	93.2							νOH_COOH_	
3165; 3159	7.5; 16.1	3167; 3157	7.7; 15.0	3173; 3157	3.0; 19.4	3166; 3156	7.3; 18.6	νCH	20a
3098	11.6	3102	12.3	3117	1.0	3100	13.5	ν_as_CH_2_	
3047	16.7	3044	28.9	3044	28.9	3041	17.6	ν_s_CH_2_	
1832	298.1							νC=O	
1765	251.4	1761	224.5	1754	207.5	1752	215.2	νC=O	
1678	267.7	1683	221.3	1683	142.0	1682	199.4	νC=C	
1656; 1650	23.2; 205.6	1659; 1649	25.1; 109.4	1655; 1649	24.2; 169.1	1657; 1652	19.4; 94.8	νCC	8a
1646; 1632	23.3; 47.8	1645; 1634	20.2; 48.2	1645; 1631	31.36; 43.6	1646; 1633	21.4; 46.4	νCC. νC=C	8b
		1589	496.8	1593	413.0	1617	507.7	ν_as_COO	
1556; 1542	128.7; 252.8	1558; 1542	140.1; 287.3	1552; 1541	107.3; 280.4	1555; 1541	130.6; 247.9	νCC	19a
1499; 1475	27.9; 64.7	1498; 1478	16.4; 13.0	1498; 1474	18.6; 57.6	1497; 1474	13.8; 68.6	βOH. νCC	19b
1487	5.2	1495	10.6	1486	8.2	1486	7.8	δ_s_CH_2_	
		1452	309.2	1428	335.1	1413	271.5	ν_s_COO	
1396; 1390	21.6; 11.2	1393; 1389	24.6; 8.8	1391; 1388	33.6; 7.2	1394; 1389	36.3; 16.9	νCC, βCH_C=C_	14
1364	9.0	1362	3.2	1360	4.2	1358	5.6	ωCH_2_	
1353; 1351	59.4; 51.1	1351; 1345	104.9; 34.7	1353; 1350	62.3; 37.9	1350; 1349	104.3; 38.4	νCC. βOH	
1344	89.9	1339	87.0	1339	14.9	1333	86.7	ωCH_2_. βCH_c=c_	
1334	142.6	1332	126.7	1333	138.3	1330	150.1	νC-OH_ar_. βOH, νCC	
1324	36.4							βOH	
1311; 1301	166.2; 358.2	1313; 1301	126.5; 409.5	1308; 1302	175.8; 236.6	1308; 1299	163.5; 404.3	βCH. νC-O	13
1214; 1213	49.7; 64.0	1214; 1211	55.8; 44.2	1214; 1209	43.6; 77.5	1215; 1212	58.9; 67.6	βCH. βOH	18b
1186	27.7	1184	142.2	1184	242.4	1187	411.9	βCH_c=c_. βCH	
1171; 1166	24.2; 352.8	1176; 1169	95.4; 256.7	1177; 1169	28.2; 58.4	1177; 1170	24.8; 64.2	βCH. βOH	18a
1124; 1123	271.4; 288.0	1124; 1121	293.8; 98.6	1138; 1129	168.4; 144.2	1123; 1122	247.4; 144.7	βCH. βOH	
1082	296.5	1085	144.1	1082	119.2	1083	100.9	ν_s_COO	
1021	28.8	1019	30.8	1018	31.6	1018	30.4	γCH_c=c_	
949; 931	2.1; 2.7	948; 930	0.4; 3.0	957; 929	3.7; 2.9	946; 928	3.7; 3.1	γCH	17a
892	0.5	894	1.5	896	0.5	895	1.9	γCH. γCH_c=c_	
879	18.6	871	9.6	877	27.6	872	11.5	ρCH_2_. αCCC	
829; 810	13.5; 46.8	825; 808	27.5; 44.9	836; 808	8.8; 29.2	821; 807	34.1; 40.1	γCH	10a
785; 771	13.6; 3.9	798; 787	7.1; 11.8	795; 782	19.0; 8.7	791; 785	13.5; 9.4	αCCC	12
		753	6.3	771	24.4	748	14.4	β_s_COO	
745	3.3							γC=O. γCH	
		738	26.8	738	13.3	735	7.0	γ_s_COO. γCH	
710; 706	13.1; 0.6	708; 701	1.2; 30.4	720; 704	3.2; 0.4	719; 708	11.4; 2.3	φCC, γCH_c=c_	4
582	62.2							γOH	
		578	27.9	565	3.9	588	2.4	β_as_COO	
530; 520	31.8; 22.4	526; 520	50.4; 4.9	528; 507	78.5; 8.7	524; 505	34.7; 10.4	αCCC	6a
496	26.6	503	2.1	507	8.7	505	10.4	αCCC	6b
467; 449	12.8; 0.1	466; 448	1.2; 4.3	466; 449	6.7; 0.1	469; 449	13.7; 0.1	φCC	16b

* fundamental modes of the phenyl ring are numbered according to Varsányi [32]; s—strong; m—medium; w—weak; v—very; sh—shoulder; ν: stretching; β: in plane deformations; γ: out of plane deformations; δ: scissoring; α: the aromatic ring in-plane bending modes; φ: the aromatic ring out-of-plane ones; τ—bending off the plane-twisting; ω—bending off the plane-fan; ρ—bending in the plane-swinging.

**Table 5:** The wavenumbers (cm^−1^) and intensities (Int) of vibrational spectra of alkali metal rosmarinates.

Rosmarinic Acid		Lithium Rosmarinate		Sodium Rosmarinate		Potassium Rosmarinate		Rubidium Rosmarinate		Cesium Rosmarinate		Assignment *	
IR	R	IR	R	IR	R	IR	R	IR	R	IR	R		
3519 m												νOH	
3456 s												νOH	
3399 s												νOH	
3313 m												νOH	
2974 w		2964 vw	2952 w	2965 vw		2963 vw		2966 w		2963 w		νCH	20a
2933 w	2946 w	2927 w		2927 vw	2931 w	2931 vw	2925 w	2928 vw		2933 vw		ν_as_CH_2_	
2857 w		2854 w		2854 vw	2855 w	2857 vw		2857 vw		2857 vw		ν_s_CH_2_	
1726 s	1727 w											νC=O	
1708 vs	1708 m											νC=O	
1645 w	1646 s	1687 s	1687 m	1687 s	1690 m	1692 s	1692 m	1692 s	1692 m	1692 s	1690 m	νC=C	
1617 s	1619 vs	1632 sh	1627 sh	1631 sh	1627 sh	1630 sh	1629 sh	1627 sh	1627 sh	1629 sh	1629 s	νCC	8a
1609 sh												νCC. νC=C	8b
		1600 vs	1606 vs	1599 vs	1608 vs	1598 vs	1606 vs	1602 vs	1606 vs	1595 vs	1606 vs	ν_as_COO	
1521 s	1525 w	1522 s	1523 vw	1522 s	1525 w	1522 s	1527 w	1522 s	1527 w	1521 s	1517 w	νCC	19a
1464 w	1478 w	1445 m	1444 w	1446 m	1447 w	1446 m	1448 w	1446 m	1447 w	1446 m	1447 w	βOH. νCC	19b
1446 w												δ_s_CH_2_	
		1412 m	1407 vw	1405 m		1403 m	1413 w	1399 sh	1407 vw	1402 m	1417 w	ν_s_COO	
1350 m		1384 w		1385 m		1385 m	1378 w				1381 w	νCC	14
		1362 m	1365 vw	1362 w	1366 vw	1362 sh	1353 w	1362 m	1355 w	1362 sh	1349 w	νCC. βOH	
1307 m	1316 w											ωCH_2_. βCH_c=c_	
1286 s	1293 w											νC-O. τCH_2_. βOH	
1260 s	1275 w	1265 vs	1264 m	1262 vs	1264 m	1260 vs	1266 m	1262 vs	1260 m	1262 vs	1266 m	νC-OH_ar_. βOH	
1232 s	1244 w											ωCH_2_	
		1183 s	1193 w	1180 s	1183 sh	1177 s	1190 sh	1177 s	1184 sh	1177 s		βCH. νC-O	13
1154 s	1154 m	1161 s	1164 m	1161 s	1162 m	1160 s	1164 m	1161 s	1162 m	1161 s	1164 m	βCH. βOH	18b
1115 m	1114 w	1116 m	1122 w	1116 m	1118 w	1116 m	1118 w	1116 s	1118 w	1116 s	1118 w	βCH. βOH	18a
1076 m	1077 vw	1069 w	1068 w	1069 w	1069 w	1069 w	1073 w	1071 m	1073 vw	1067 m	1075 w	βCH_c=c_. βCH	
		1034 w	1041 vw	1032 w	1033 vw	1032 w		1037 w	1027 vw	1034 w	1037 vw	βCH. βOH	
981 sh												γCH_c=c_	
973 m	972 vw	976 m	981 w	974 m	975 m	974 m	979 w	976 m	977 w	976 m	979 w	γCH	17a
916 vw	917 w											νOH_COOH_	
851 w	852 w	853 m	853 w	853 w	853 w	853 m	857 w	854 m	857 w	854 m	861 w	γCH. γCH_c=c_	5
819 m	807 w	810 m	813 w	810 m	805 vw	810 m	813 w	812 m	813 w	812 m	811 w	γCH	10a
790 w	792 w											βC=O	
782 w		783 w	788 w	782 w	788 w	782 w	784 w	783 w	786 w	782 w	786 w	αCCC	12
682 w												γC=O. γCH	
642 m	641 w											γOH_ar_	
		736 vw	751 w	735 vw	742 vw	738 vw	738 w	738 vs	730 w		728 w	β_s_COO	
594 w		715 vw	718 vw	718 vw	722 w	717 vw	724 w	719 vw	718 vw	718 vw	713 vw	γ_s_COO. γCH	
		593 w	597 w	592 w	591 w	591 w	597 vw	592 w	589 w	592 w	597 w	αCCC	6a
		565 w	568 w	564 vw		564 vw	570 vw	574 vw	573 vw	563 vw	568 vw	β_as_COO	
531 w	526 vw	525 w	524 vw	521 w	524 vw	521 w	526 vw	523 w	522 w	521 w	526 w	αCCC	6b
465 w	458 w	448 w	454 w	456 w		456 w	454 w	455 w	456 vw	457 w		φCC	16b

* Fundamental modes of the phenyl ring are numbered according to Varsányi [32]; s—strong; m—medium; w—weak; v—very; sh—shoulder; ν: stretching; β: in plane deformations; γ: out of plane deformations; δ: scissoring; α: the aromatic ring in-plane bending modes; φ: the aromatic ring out-of-plane ones; τ—bending off the plane-twisting; ω—bending off the plane-fan; ρ—bending in the plane-swinging.

**Table 6:** The experimental and theoretical (6-311+G(d.p)) chemical shifts (δ/ppm) of protons and carbons in NMR spectra of rosmarinic acid and its alkali metal salts in DMSO solutions.

	Rosmarinic acid			Rosmarinates							
Atom *	Exp.		Calc.	RA-Li		RA-Na		RA-K		RA-Rb	RA-Cs
	[8]			Exp.	Calc.	Exp.	Calc.	Exp.	Calc.	Exp.	Exp.
C1	127.6	125.36	132.75	125.48	132.86	125.48	133.07	125.45	133.20	125.45	125.41
C2	114.5	113.26	115.54	114.73	115.47	114.73	115.19	114.68	114.88	114.21	114.62
C3	146.8	145.93	150.95	146.07	151.38	146.00	151.19	146.09	150.95	145.88	146.15
C4	149.7	148.63	153.46	148.86	153.02	148.73	152.75	148.73	152.25	148.71	148.85
C5	116.2	116.70	117.72	116.81	118.13	116.70	118.08	116.70	117.76	116.73	116.77
C6	123.1	121.58	132.56	120.90	131.59	120.82	131.45	120.57	131.20	121.05	120.58
C7	147.6	144.94	156.96	144.47	154.15	144.39	152.48	144.39	153.42	144.93	144.47
C8	115.2	115.40	113.42	115.58	116.60	115.49	117.16	115.47	116.75	115.50	115.54
C9	168.5	165.92	171.03	166.33	170.56	166.30	170.56	166.32	170.39	166.16	166.38
C1′	129.4	127.29	132.45	129.87	137.31	129.83	138.78	129.88	139.68	128.99	129.88
C2′	117.5	115.77	119.00	116.17	119.55	116.06	118.98	116.00	119.11	116.03	116.04
C3′	146.1	145.60	149.36	145.05	148.07	144.98	147.81	144.98	147.56	144.97	145.03
C4′	145.2	144.02	150.28	143.71	148.58	143.63	148.13	143.61	148.28	143.78	143.66
C5	116.5	114.90	119.00	115.17	119.10	115.11	119.10	115.23	118.83	115.16	115.29
C6′	121.8	120.04	127.96	119.75	126.75	119.67	126.49	119.60	126.38	119.80	119.62
C7′	37.9	36.11	41.07	37.23	35.83	37.25	36.02	37.26	35.83	36.84	37.25
C8′	74.8	72.82	144.95	75.93	144.37	75.95	143.79	76.11	144.91	74.89	76.21
C9′	167.4	170.83	165.08	172.88	183.49	173.07	179.28	172.51	175.57	172.11	172.52
H2	6.88	7.06	7.65	7.05	7.65	7.05	7.61	7.05	7.50	7.06	7.06
H3		9.64	5.47		5.41		5.35		5.30		
H4		8.79	4.99		4.86		4.84		4.91		
H5	6.61	6.77	7.00	6.76	6.95	6.76	6.98	6.74	7.03	6.76	6.74
H6	6.79	7.01	7.03	6.92	7.04	6.92	7.05	6.90	6.96	6.95	6.90
H7	7.39	7.47	7.74	7.38	7.98	7.38	7.84	7.38	7.73	7.41	7.39
H8	6.11	6.24	6.54	6.19	6.42	6.19	6.40	6.18	6.38	6.21	6.19
H2′	6.59	6.68	7.03	6.69	6.85	6.68	6.81	6.68	6.76	6.68	6.68
H3′		8.73	4.50		4.35		4.30		4.30		
H4′		9.16	5.27		5.17		5.06		4.98		
H5′	6.54	6.64	6.97	6.61	6.97	6.61	6.96	6.60	6.94	6.62	6.60
H6′	6.47	6.53	7.05	6.50	6.91	6.50	7.02	6.49	7.00	6.51	6.49
H7′	2.85	2.99	4.34	3.05	4.81	3.03	4.83	3.01	4.10	3.02	3.00
H7′’	2.93	2.90	3.56	2.77	4.30	2.78	4.24	2.77	5.31	2.81	2.77
H8′	5.03	5.03		4.90		4.89		4.86		4.93	4.85
H9′		12.99	5.96								

* the numbering of atoms according to Figure 1.

**Table 7:** The results of kinetics studies of photolysis of direct rosmarinic acid and its alkali metal salts.

Substance	k^1^/min^−1^	t_1/2_^2^/min	t_1/2_/h	pH before Irradiation	pH after Irradiation
RA-Cs	1.7 10^−3^	408	6.8	6.50	6.25
RA-Rb	0.9 10^−3^	770	12.8	5.09	5.49
RA-K	1.0 10^−3^	693	11.6	6.18	6.36
RA-Na	2.2 10^−3^	315	5.3	6.09	6.17
RA-Li	1.3 10^−3^	533	8.9	6.52	6.24
RA	0.8 10^−3^	866	14.4	4.23	4.92

^1^ k—rate constant; ^2^ t_1/2_—half time of the process.

## Supplementary Materials

The following are available online, Figure S1: The correlation between calculated and literature data of bonds lengths and angles.
