# Supplementary figures and images for: Molecular Structure and Antioxidant Properties of Alkali Metal Salts of Rosmarinic Acid. Experimental and DFT Studies
Source: Molecules. 2019 Jul 21;24(14):2645. doi: 10.3390/molecules24142645 (PMC6680818; doi:10.3390/molecules24142645)

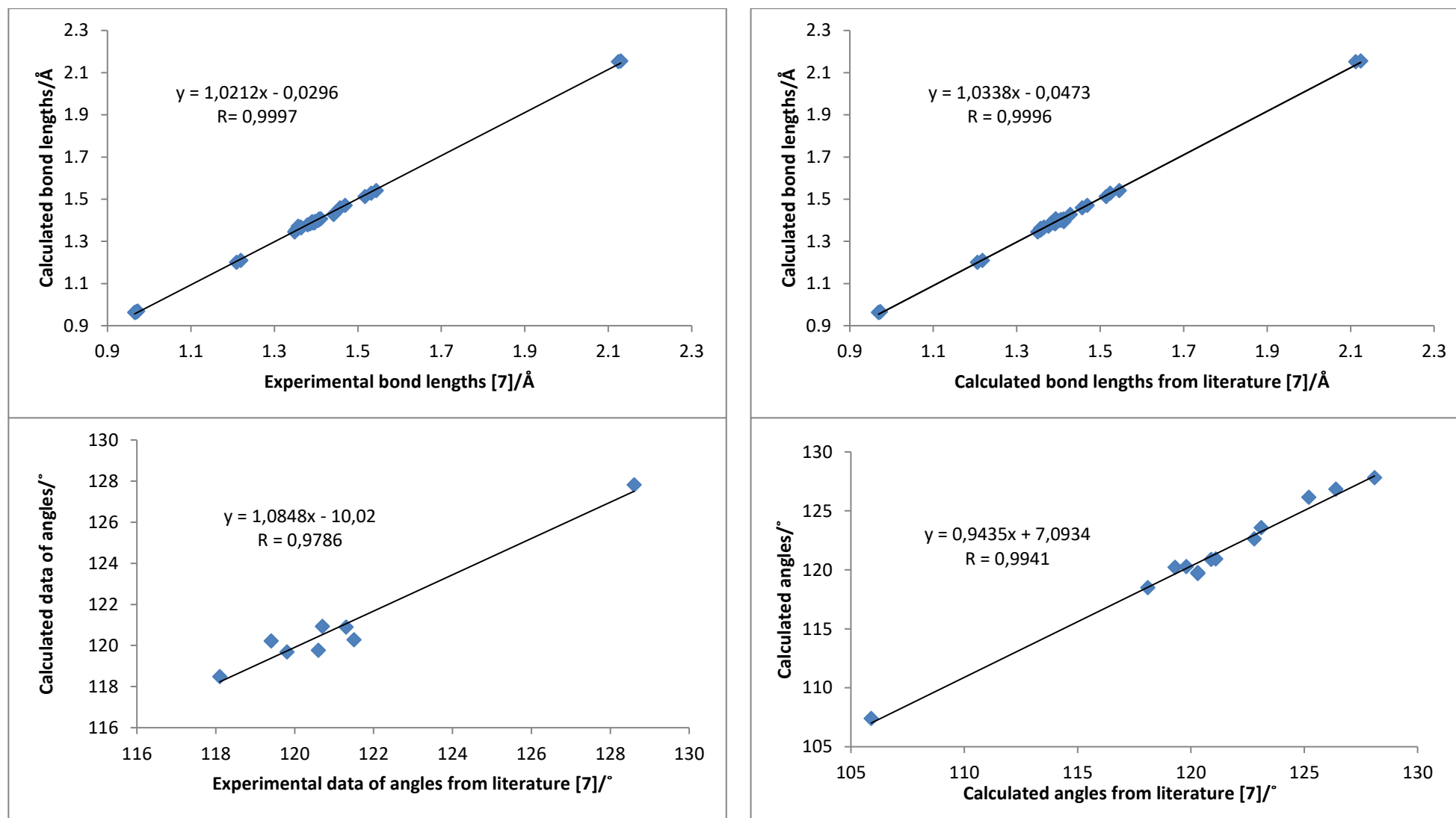

**Figure S1.** The correlation between calculated and literature data of bonds lengths and angles

Supplement: Supplementary file 1 [file molecules-24-02645-s001.pdf]
